# Addiction to *Runx1* is partially attenuated by loss of p53 in the Eμ-Myc lymphoma model

**DOI:** 10.18632/oncotarget.8554

**Published:** 2016-04-02

**Authors:** Gillian Borland, Anna Kilbey, Jodie Hay, Kathryn Gilroy, Anne Terry, Nancy Mackay, Margaret Bell, Alma McDonald, Ken Mills, Ewan Cameron, James C. Neil

**Affiliations:** ^1^ Molecular Oncology Laboratory, Institute of Infection, Immunity and Inflammation, University of Glasgow, Glasgow, UK; ^2^ School of Veterinary Medicine, University of Glasgow, Glasgow, UK; ^3^ Centre for Cancer Research and Cell Biology, Queen's University Belfast, Belfast, UK

**Keywords:** Runx1, lymphoma, myc, oncogene addiction

## Abstract

The *Runx* genes function as dominant oncogenes that collaborate potently with Myc or loss of p53 to induce lymphoma when over-expressed. Here we examined the requirement for basal Runx1 activity for tumor maintenance in the Eμ-Myc model of Burkitt's lymphoma. While normal *Runx1^fl/fl^* lymphoid cells permit mono-allelic deletion, primary Eμ-Myc lymphomas showed selection for retention of both alleles and attempts to enforce deletion *in vivo* led to compensatory expansion of p53^null^ blasts retaining *Runx1*. Surprisingly, *Runx1* could be excised completely from established Eμ-Myc lymphoma cell lines *in vitro* without obvious effects on cell phenotype. Established lines lacked functional p53, and were sensitive to death induced by introduction of a temperature-sensitive p53 (Val135) allele. Transcriptome analysis of *Runx1*-deleted cells revealed a gene signature associated with lymphoid proliferation, survival and differentiation, and included strong de-repression of recombination-activating (*Rag)* genes, an observation that was mirrored in a panel of human acute leukemias where *RUNX1* and *RAG1,2* mRNA expression were negatively correlated. Notably, despite their continued growth and tumorigenic potential, Runx1^null^ lymphoma cells displayed impaired proliferation and markedly increased sensitivity to DNA damage and dexamethasone-induced apoptosis, validating Runx1 function as a potential therapeutic target in Myc-driven lymphomas regardless of their p53 status.

## INTRODUCTION

*Runx1* encodes a transcription factor that plays a vital role in development of the haematopoietic system [1]. It belongs to a three-membered family of mammalian gene products that bind a common DNA target sequence by virtue of the conserved Runt domain and share a common heterodimeric binding co-factor, CBFβ [2, 3]. Like their Drosophila homologue, Runt, the Runx proteins function as transcriptional regulators and are capable of activating or repressing target promoters through the recruitment of co-activators or co-repressors [4]. The *RUNX1* (*AML1*) and *CBFB* genes are among the most commonly involved in human leukemias where they are affected by chromosomal translocations that frequently generate fusion oncoproteins [5].

Evidence that simple over-expression of any of the *Runx* gene family members can drive oncogenesis emerged first from mouse models, where it was shown that all three genes can act as targets for murine leukemia virus (MLV) insertional mutagenesis and transcriptional activation in lymphoma. Common targets in the Eμ-Myc lymphoma model include *Runx1* and *Runx3* [6], while all three members of the *Runx* family were identified as activation targets in CD2-MYC T-cell lymphomas [7–9]. The potent oncogenic effect of combining Myc and Runx over-expression is emphasised further in retroviral acceleration of lymphoma onset in *Runx2* transgenic mice which frequently entails activation of c-*Myc* or N-*Myc* [10], while compound transgenic mice over-expressing Myc and Runx genes in the T or B-cell compartment display very rapid tumor onset [10–12]. However, the *Runx* family are not merely cofactors for Myc oncogenesis; CD2-*Runx2* transgenic mice display dose-dependent predisposition to lymphoma [11, 13] and strongly synergistic lymphoma development in combination with other oncogenes such as *Pim-1* and *v-Myb*, as well as with loss of p53 [10]. Notably, the combination of *Runx2* and *Myc* oncogenes appears to overcome the need for mutational inactivation of p53 [14] despite the fact that both genes can trigger the p53 pathway and collaborate with p53 loss when over-expressed individually [15, 16].

In contrast to this catalogue of evidence of dominant oncogenic activity in lymphomagenesis, *Runx1* deficient cells in chimeric mice develop T-cell lymphomas after treatment with ENU [17], suggesting that loss of *Runx1* activity can also predispose to lymphoid malignancy. A similar dichotomy of observations exists for *RUNX1* in human haematopoietic cancers. *RUNX1* is among the most over-expressed genes in childhood ALL [18] and is highly amplified in a poor prognostic B-ALL subgroup [19] while presumptive loss-of-function *RUNX1* mutations have been observed in a small proportion of T-ALLs where network analysis further implicated *RUNX1* as a candidate tumor suppressor [20]. More extensive evidence of a tumor suppressor role for RUNX1 has come from myeloid malignancies where loss of function mutation is frequently observed in AML, and underlies familial platelet disorder with predisposition to AML [21, 22].

While the lymphomagenic effects of Runx over-expression have been amply demonstrated, the requirement for basal gene expression in tumor maintenance is an important and potentially far-reaching question that has been much less well investigated. In this study we tested the effects of ablating the endogenous *Runx1* gene in the well-characterised Eμ-Myc lymphoma model system [23] where ectopic expression of *Runx1* is known to drive lymphomagenesis [12]. We show that primary Eμ-Myc lymphomas have an increased requirement for *Runx1*, while this dependency is reduced, but not eliminated, in end-stage p53-deficient lymphoma cell lines. Our findings shed light on the paradoxical observation that *Runx1* deficiency can also predispose to lymphoma but more importantly validate Runx1 function as a therapeutic target in p53 wild-type or mutant lymphomas.

## RESULTS

### Addiction to Runx1 in primary Eμ-Myc lymphoma cells is attenuated in established cell lines

Eμ-Myc mice develop lymphomas with highly variable onset (average 30 weeks) during which they acquire a range of secondary mutations in the Cdkn2a-p53 pathway [24]. To achieve more homogeneous tumor onset and facilitate tracking of p53 loss along with *Runx1* deletion, we crossed these mice to a *Trp53*^+/−^ background. Eμ-Myc/p53^+/−^ mice succumb to B-cell lymphomas within a much narrower time window [25]. These mice were further crossed to generate Mx1Cre/*Runx1^fl/fl^* cohorts in which we could examine the ability of lymphoma cells to survive deletion of the endogenous *Runx1* gene.

**Figure 1:**
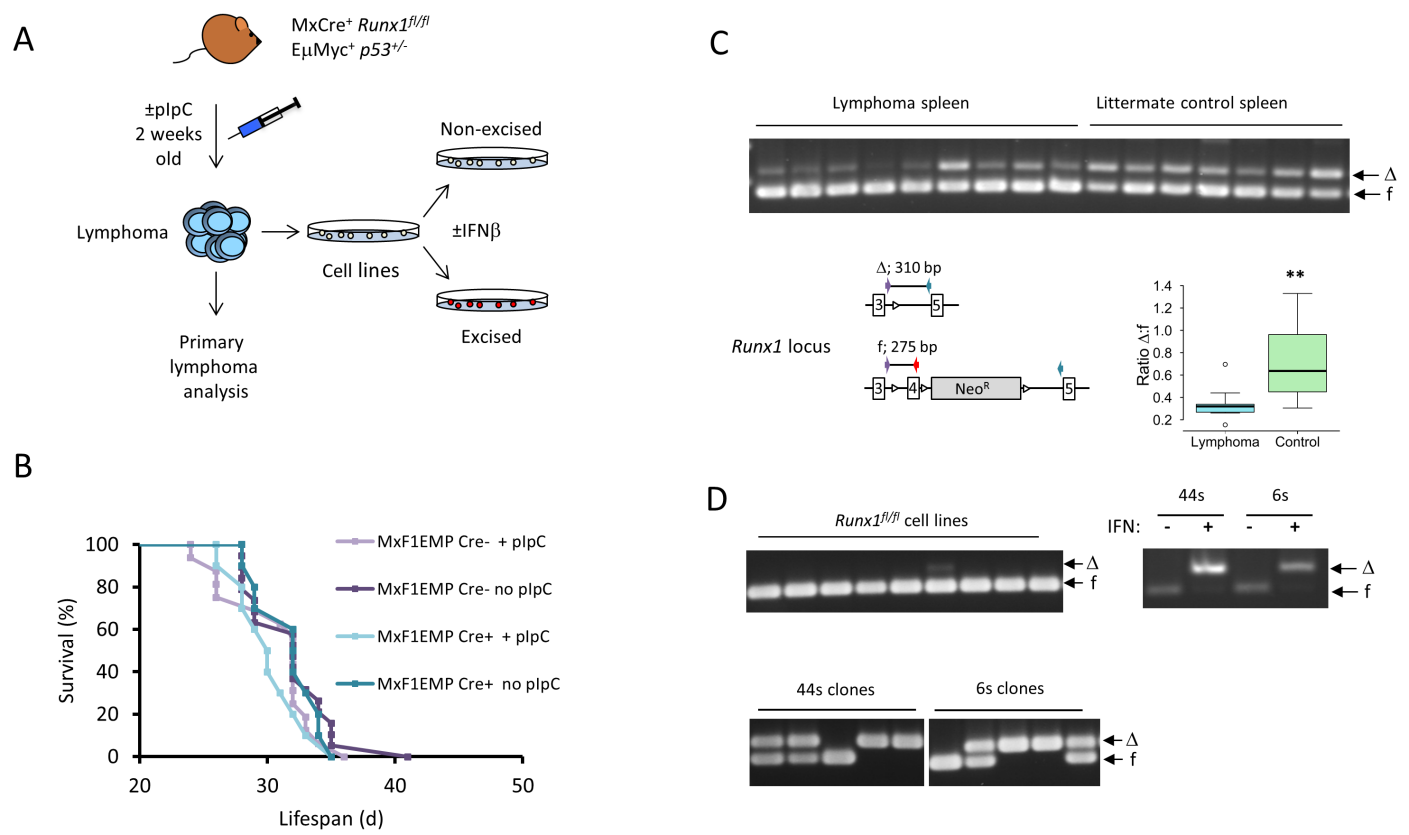
Eμ-Myc lymphomas strongly resist deletion of *Runx1 in vivo* but not *in vitro* **A.** Outline experimental design Lymphomas derived from Mx1Cre^+^/*Runx1^fl/fl^*/Eμ−Myc/*p53^+/−^* mice were analysed and also used to establish cell lines that could be treated *in vitro* with IFNβ to induce *Runx1* excision. **B.** Survival curve for *Runx1^fl/fl^*/Eμ−Myc^+^/*p53^+/−^* mice with or without the Mx1Cre transgene and with or without pIpC treatment to excise *Runx1*. Statistical analysis by Wilcoxon Rank Sum test showed no significant difference in survival between the groups of mice. **C.** Upper panel : *Runx1* excision PCR on genomic DNA derived from lymphoma spleen tissue of pIpC-treated Mx1Cre^+^/*Runx1^fl/fl^*/Eμ-Myc^+^/*p53^+/−^* mice (lymphoma spleen) and age-matched Eμ-Myc^−^ (lymphoma-free) littermate controls (littermate control spleen). Arrows indicate *Runx1*-floxed (f) and *Runx1*-deleted (Δ) bands. The panel below left shows a diagram of the multiplex PCR for detection of deleted (Δ) and floxed (f) Runx1. The cartoon shows loxP sites flanking exon 4 in the floxed allele before and after excision, location of primers (colored arrows) and size of PCR products. The panel below right shows the ratio of excised:non-excised band intensity determined by densitometry for the *Runx1* excision PCR samples shown in 1C. Boxplot shows the distribution of all the Δ:f ratios with the box representing the 1^st^ to 3^rd^ quantiles (Q1 to Q3) and the midline representing the median. Whiskers represent the smaller of the most extreme data point, or 1.5x the Q1-Q3 interquantile range. Asterisks denote statistical significance, with *p* = 0.008 **D.** The upper left panel shows *Runx1* excision PCR on genomic DNA from a series of independent cell lines derived from Mx1Cre^+^/*Runx1^fl/fl^*/Eμ-Myc^+^/*p53^+/−^* lymphomas. The right panel shows *Runx1* excision PCR on genomic DNA from samples of 44s and 6s Mx1Cre^+^/*Runx1^fl/fl^*/Eμ-Myc^+^/*p53^+/−^* cell lines treated with IFNβ to excise *Runx1* or with vehicle control; cell samples were taken 2 days after the start of IFNβ treatment. The lower panel shows *Runx1* excision PCR on genomic DNA from single cell clones derived from the 44s and 6s Mx1Cre^+^/*Runx1^fl/fl^*/Eμ-Myc^+^/*p53^+/−^* cell lines; cells were treated with a sub-optimal dose of IFNβ to induce partial excision of *Runx1* and single cell cloned as detailed in Materials and Methods. Arrows indicate *Runx1*floxed (f) and *Runx1*-deleted (Δ) bands.

Surprisingly, we found no significant difference in the rate of onset of lymphoma in Eμ-Myc/p53^+/−^/*Runx1^fl/fl^* mice with active Cre recombinase (Figure 1A, 1B), initially suggesting that Runx1 loss had no effect on tumor onset. However, PCR analysis (Figure 1C) of tumor-bearing spleens showed that the intact *Runx1* allele was strongly retained in the primary tumors, even in pIpC-treated mice. Spleens from mice with end-stage disease, which were markedly enlarged due to lymphoma expansion, showed levels of *Runx1* deletion significantly lower than normal splenic lymphoid cells from age-matched Mx1Cre^+^/Runx1*^fl/fl^* mice without the Eμ-Myc oncogene, indicating an increased rather than a decreased requirement for *Runx1* in primary lymphoma cells (Figure 1C, upper panel and lower right panel).

While the cell lines derived from these lymphomas almost uniformly retained the functional active *Runx1* allele on initial establishment in culture (Figure 1D, upper left panel), they were able to survive excision of both wild-type alleles after treatment with IFNβ *in vitro* (Figure 1D, upper right panel). Moreover, single cell cloning of these lines after low dose IFNβ readily generated subclones with 0, 1 or 2 intact *Runx1* alleles (Figure 1D, lower panel). Using these lines we validated the direct PCR assay as a consistent measure of the proportion of excised to non-excised allele. In this assay, the observed ratio of excised to non-excised allele is proportionate to input and independent of DNA concentration (Figure S1A, S1B).

### Permissiveness for Runx1 deletion is lineage-dependent

**Figure 2:**
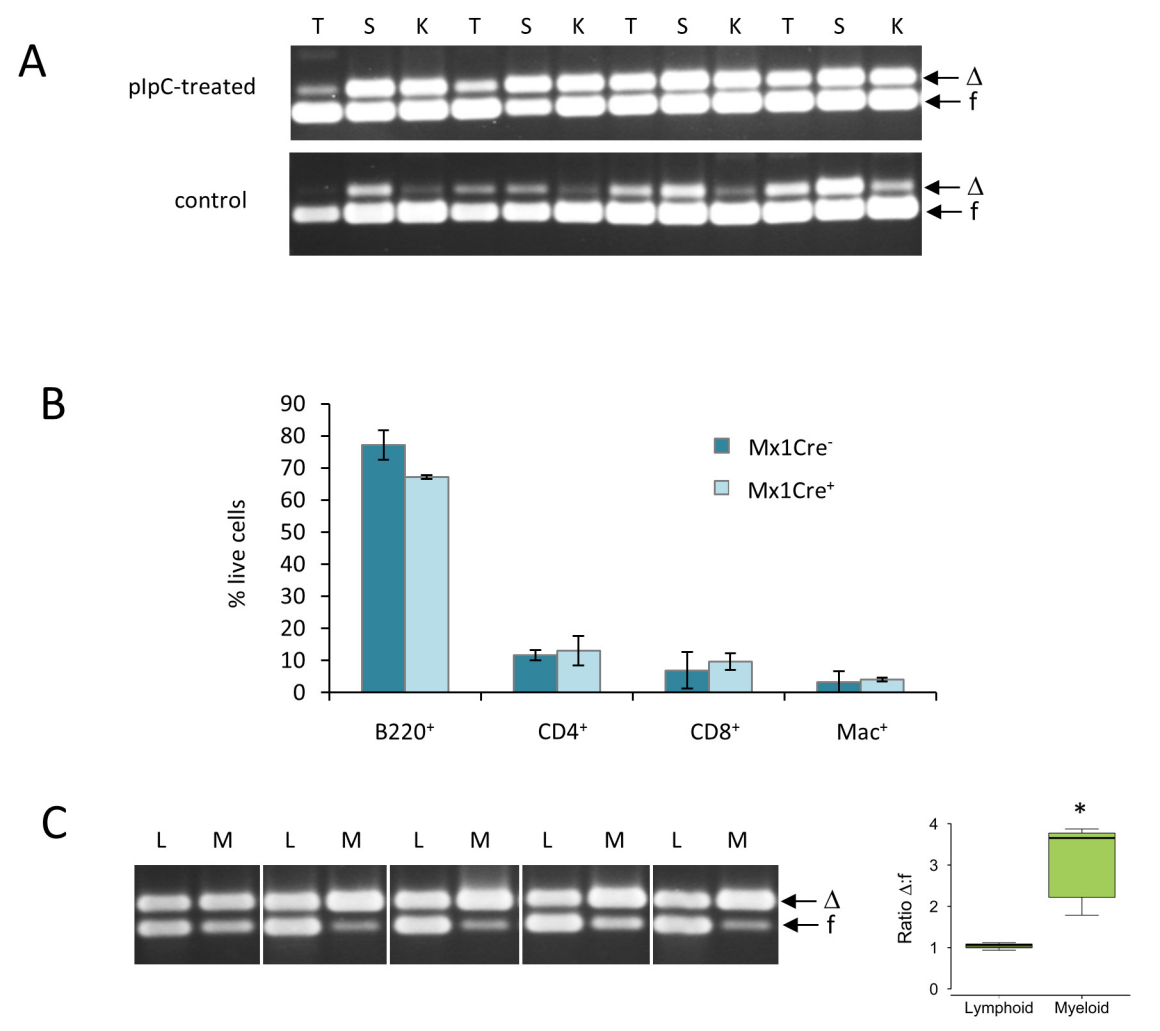
Healthy tissues display lineage-specific differences in permissiveness for *Runx1* deletion **A.** *Runx1* excision PCR analysis of genomic DNA from whole thymus (T), spleen (S) and kidney (K) of non-lymphoma-bearing adult *Runx1^fl/fl^* mice treated (upper panel) or untreated (lower panel) with pIpC to induce Mx1Cre expression. Arrows indicate *Runx1*floxed (f) and *Runx1*-deleted (Δ) bands. **B.** Flow cytometric analysis of spleen cell populations in pIpC-treated Mx1Cre^−^ and Mx1Cre^+^ Eμ-Myc^−^ healthy littermate control mice. Plot shows mean ± SD for measurements from 3 mice in each group; no statistically significant differences between the Mx1Cre^+^ and Mx1Cre^−^ cell populations were found. **C.** *Runx1* excision PCR analysis of genomic DNA from sorted lymphoid (B220^+^ or CD3^+^ cells; L) and myeloid (Mac1^+^; M) cells from non-lymphoma-bearing spleens of 5 pIpC-treated adult *Runx1^fl/fl^* mice. Boxplot shows the distribution of all the Δ:f ratios as described in Figure 1. Asterisk indicates that the difference is statistically significant (*p* = 0.01).

In accord with previous reports [26], analysis of non-tumor bearing mice revealed lineage-dependence in permissiveness for *Runx1* deletion. Healthy tissues showed a marked difference in the levels of spontaneous and enforced deletion of *Runx1*, with thymus consistently more resistant than kidney or spleen. However, even the permissive tissues showed at most 50% deletion (Figure 2A). Mono-allelic deletion in *Runx1^fl/fl^* cells has been reported previously in *Runx1*-dependent tissues from vav-Cre*Runx1^fl/fl^* mice [26]. These results suggest that a strong homeostatic process selects for cells that retain at least one copy of *Runx1*. Consistent with this hypothesis, the cell type composition of spleen showed remarkably little change after enforced *Runx1* deletion (Figure 2B). However, fractionation of spleen cells into lymphoid (B220^+^ or CD3^+^) or myeloid (Mac1^+^) confirmed the relative permissiveness of the myeloid compared to the lymphoid compartment for deletion of Runx1 [26] (Figure 2C).

### Enforced deletion of Runx1 promotes the outgrowth of p53 null cells in Eμ-Myc/p53+/− lymphomas

**Figure 3:**
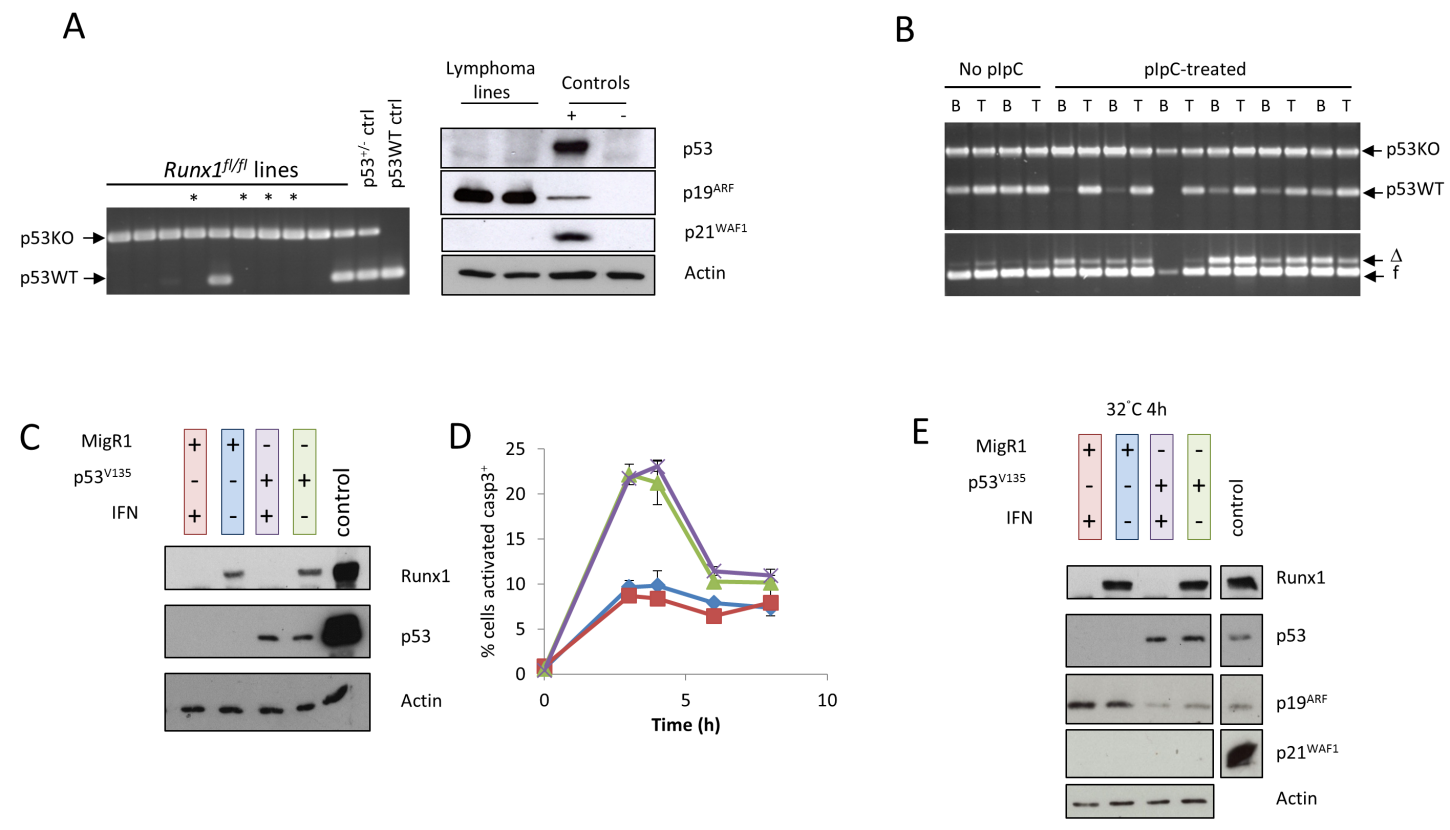
Loss of p53 precedes permissiveness for *Runx1* deletion during establishment of lymphoma cell lines **A.** Left panel: p53 allele PCR on genomic DNA from a series of independent cell lines derived from lymphomas in Mx1Cre^+^/*Runx1^fl/fl^*/Eμ-Myc^+^/*p53^+/−^* mice; arrows indicate p53^null^ (p53KO) and wild type (p53WT) alleles in control samples for p53^wt/wt^ and p53^+/−^; asterisks indicate cell lines established from pIpC-treated mice. Right panel: western blot analysis of total protein extracted from two established and independent lymphoma cell lines derived from lymphomas in Mx1Cre^+^/*Runx1^fl/fl^*/Eμ-Myc^+^/*p53^+/−^* mice that did not receive pIpC treatment. Extracts were probed with antibodies to p53, p19^ARF^ and p21^WAF1^. Actin was used as a loading control. Positive controls are listed in Materials & Methods and used in all subsequent analyses. **B.** Upper panel shows p53 allele PCR on genomic DNA from sorted blast cells (B) or whole tissue (T) from lymphoma-bearing spleens of untreated and pIpC-treated Mx1Cre^+^/*Runx1^fl/fl^*/Eμ-Myc^+^/*p53^+/−^* mice, while the lower panel shows *Runx1* excision PCR on the same DNA samples. Blast cells were sorted using CD45/SSC on B220^+^ cells as detailed in Materials and Methods. Arrows indicate p53^null^ (p53KO) and wild type (p53WT) alleles, and *Runx1*floxed (f) and *Runx1*-deleted (Δ) bands. **C.** Cells transduced with vector expressing temperature-sensitive p53^V135^ or the MigR1 control vector were treated with IFNβ to excise endogenous *Runx1,* and expression of p53 and Runx1 examined by western blot analysis. **D.** Paired Runx1^+^ and Runx1^null^ p53 addback cell lines treated with 5J/m^2^ UV were grown at 32^°^C to activate temperature sensitive p53 and stained for intracellular activated caspase 3 after 3, 4, 6 and 8h incubation. The percentage of cells expressing activated caspase 3 was determined by flow cytometry. Line colors are indicated by the color-coding of the western blots samples in 3C. **E.** Western blot analysis of total protein extracted from the experiment as shown in (D) 4h after UV treatment. Extracts were probed as before and loading confirmed by actin expression.

Established lymphoma cell lines were found to have lost the wild-type *Trp53* allele in most cases and displayed consistent de-repression of Cdkn2a/p19^Arf^ [24] (Figure 3A, S2A). We therefore considered whether the contrasting behaviour of primary lymphomas and established cell lines with regard to retention of Runx1 was a consequence of loss of the wild-type *Trp53* allele. To address this hypothesis further and examine the temporal order of events *in vivo*, we analysed the minor blastic fraction of cells from end-stage lymphoma-bearing spleens, a procedure that has been shown to enrich for the most rapidly proliferating cells within the tumor [27]. The fractionated blasts displayed strong enrichment for cells that had lost the wild-type *Trp53* allele (Figure 3B). The fact that most lymphoma blast cells retained at least one *Runx1* allele indicated that loss of p53 was an earlier event that preceded permissiveness for *Runx1* loss, a conclusion reinforced by findings on newly established cell lines (Figure 1D, upper left panel). Enrichment for p53-deleted blasts was observed most strongly in pIpC-treated mice, suggesting that attempts to enforce deletion of *Runx1* may have perturbed the tissue sufficiently to create a permissive niche for outgrowth of bystander p53^−/−^ blasts. These results further emphasise the importance of *Runx1* for survival of Eμ-Myc lymphoma cells *in vivo,* even though it becomes dispensable in established cell lines *in vitro.*

The hypothesis that Runx1 is required to counteract the growth suppressive effects of p53 was tested by transduction of a p53 null Eμ-Myc/*Runx1^fl/fl^* lymphoma cell line (3s) with a temperature-sensitive p53 allele (Val135). After transduction, interferon treatment was used to produce matched pairs of cell lines with or without endogenous Runx1 that could be tested for sensitivity to p53-induced death. However, the p53 “add-back” cells died by apoptosis at the permissive temperature (32^°^C) regardless of their Runx1 status or exposure to UV irradiation (Figure 3C, 3D, S2B). The functional integrity of the p53 pathway was maintained with respect to down-regulation of Cdkn2a/p19^ARF^ in response to temperature shift but the major target for p53-mediated growth arrest, Cdkn1a/p21^WAF1^, was undetectable in these cells (Figure 3E), suggesting a partial functional deficit. As it has been reported that p53 can affect Runx1 expression in T-cells [28], we also examined Runx1 expression in 3s cells, but saw no evidence of modulation by p53 (Figure 3E).

### Runx1 deficient cells display down-regulation of genes involved in growth and proliferation along with de-repression of *Rag* genes

Changes in gene expression resulting from deletion of *Runx1* were examined using Affymetrix gene expression microarrays (Affymetrix GeneChip MTA 1.0). Three biological replicates of the 3s cell line were compared. Possible confounding effects of IFNβ treatment and Cre recombinase induction were controlled by comparison with a phenotypically matched Eμ-Myc lymphoma cell line (30s) of the same genotype apart from the non-deletable *Runx1^wt/wt^* allele. Treatment with IFNβ had no effect on Runx1 expression in these cells (Figure S3A, S3B).

**Figure 4:**
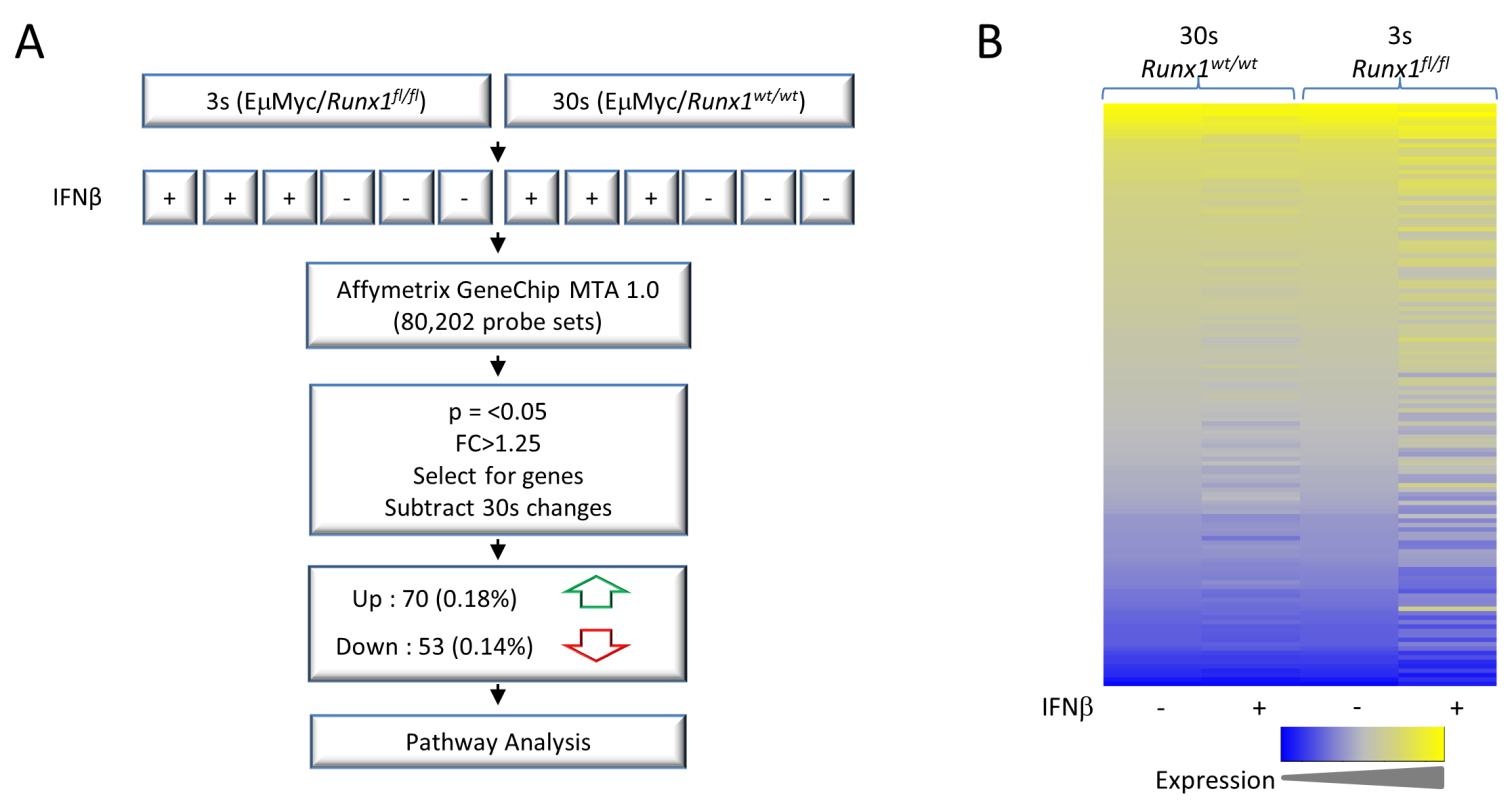
Gene expression microarray analysis of E**μ**-Myc lymphoma cells before and after *Runx1* excision **A.** Process overview. 3s+ and 30s+ cell lines derived from Mx1Cre^+^/*Runx1^fl/fl^*/Eμ-Myc^+^/*p53^+/−^* mice were treated with IFNβ, excising Runx1 from 3s cells (*Runx1^fl/fl^*) but not 30s controls where interferon responses induce Cre expression but deletion does not occur on the *Runx1^wt/wt^* background (see also Figure S3). RNA was extracted and gene expression examined by Affymetrix microarray. Significantly changed genes were selected by *p*-value and fold-change and a subset that were also significantly changed in 30s cells were subtracted to exclude non-Runx1 related changes. This gene list was then used in downstream analyses such as pathway analysis. **B.** Heat map of all genes significantly changed in 3s cells, defined as *p* < 0.05 and fold change > |1.25|. Shown is raw intensity for 3s and 30s cells. Low expression is shown in blue and high expression in yellow. 30s gene expression was normalised to 3s control (−IFNβ) expression.

**Figure 5:**
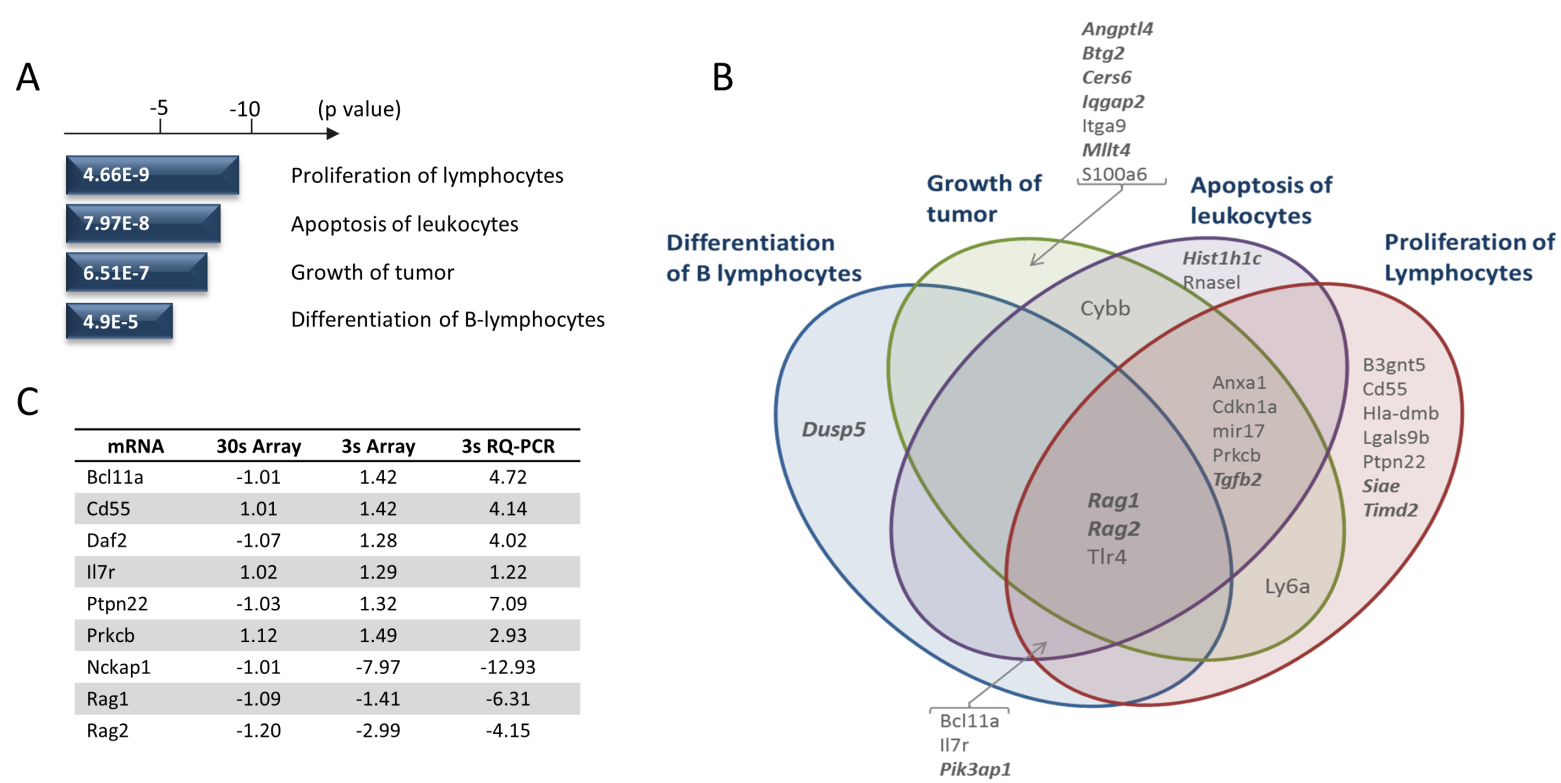
Gene expression changes specific for Runx1 deletion in Eμ-Myc lymphoma cells **A.** Ingenuity Pathway Analysis of the most changed genes (Figure 4) revealed significant enrichment for genes involved in the processes depicted. *P*-values are shown for top sub-processes in relevant categories. **B.** Venn diagram showing the overlap of the gene signatures for the significant processes in **A.** Genes in bold italics show increased expression on loss of *Runx1,* while the remainder are down-regulated. **C.** Validation of key target genes contributing to the Runx1 deletion signatures. Fold changes estimated by microarray and RQ-PCR are shown alongside comparable array values for the 30s control cells.

Many genes showed changes in expression specific to the *Runx1^fl/fl^* genotype. To focus on the most prominent changes we applied a statistical significance threshold of *p* = < 0.05 and a fold change cut-off of 1.25. After subtraction of genes significantly changed in the 30s control, this left 123 genes of which 70 were up-regulated and 53 down-regulated on excision of *Runx1* (Figure 4A). As can be seen from the heat map in Figure 4B, the control 30s cells showed relatively modest changes after IFNβ treatment compared to *Runx1^fl/fl^* 3s cells. The gene set specific for Runx1 loss was subjected to Ingenuity Pathway Analysis which revealed significant associations with cancer and in particular with proliferation, apoptosis and differentiation of lymphocytes (Figure 5A). The direction of changes on deletion indicated that Runx1 is acting to sustain proliferation and survival while impeding differentiation in these cells (Figure 5B). *Rag1* and *Rag2* were common components of all four pathway clusters (Figure 5B) and were among the most strongly de-repressed genes in *Runx1*-deleted cells. Validation of the changes in *Rag* genes and other signature genes by quantitative real-time PCR confirmed the array findings and revealed larger fold changes in most cases, consistent with the precision but systematic underestimation of differences by this methodology [29] (Figure 5C).

As recent studies have defined Runx1/RUNX1 gene expression signatures in other contexts, we examined our array data for similar changes in the key genes. An RNA-Seq study of normal haematopoietic stem and progenitor cells using the same *Runx1^fl/fl^* allele revealed a ribosome biogenesis gene expression signature associated with loss of Runx1 [30]. However, we saw no significant difference in these genes. A subset of the most changed genes from the signature gene set is shown in Figure S3C. Nor did we note any obvious change in cell size or morphology in *Runx1*-excised Eμ-Myc lymphoma cell lines (Figure S3D). We also examined a mitotic checkpoint gene signature that was reported in Kasumi/AML cells after knockdown of RUNX1 [31]. The genes shown in Figure S3E were all significantly downregulated in Kasumi knockdown cells but were mostly unchanged in *Runx1^null^* Eμ-Myc lymphoma cells. Only *Nek6* was significantly down-regulated while two of the genes showed a significant increase (*Nek2*, *Bub1b*).

### Negative correlation of RUNX1 and RAG mRNA expression in a human leukemia panel

**Figure 6:**
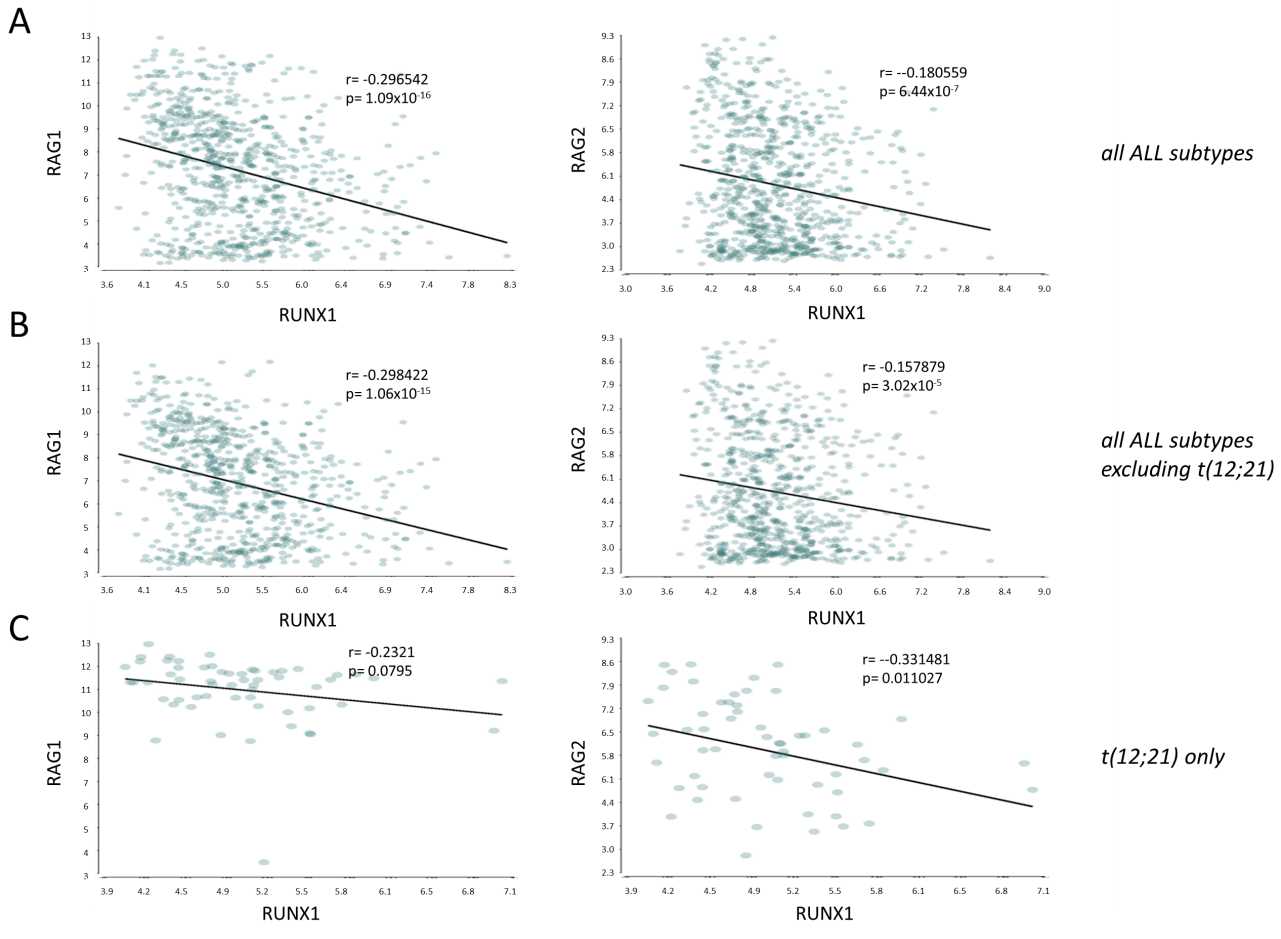
*RUNX1* mRNA expression is negatively correlated with *RAG1* and *RAG2* in a large panel of human ALLs Pearson correlation plots of gene expression (probeset intensities) of *RUNX1 vs RAG1* or *RAG2* in **A** all ALL subtypes within the MILE database (ref [33]) (*n* = 750), **B.** all ALL subtypes with the exception of the t(12;21)/*ETV6-RUNX1* subset (*n* = 692), **C.** the t(12;21) translocation only (*n* = 58). Plots show *p*-value and r-value.

Aberrant RAG activity has recently been reported as a major source of cancer driver mutations in *TEL-RUNX1* t(12;21) B-cell leukemias [32], leading us to consider whether there may be a wider role for RUNX1 in RAG mis-regulation in human leukemia/lymphomas. Using Partek Genomics Suite 6.6 we examined the relationship between expression of *RUNX1* and *RAG1/RAG2* expression in a panel of acute and chronic leukemias from the MILE study (Microarray Innovations in LEukemia), a global microarray study comprising gene expression analysis of > 4000 patients [33]. As shown in Figure S4, *RUNX1* mRNA expression was relatively uniform compared to the *RAG* genes that displayed markedly higher expression in ALLs compared to normal tissues and myeloid leukemias. Also, expression of *RAG1* was significantly higher in t(12;21) ALLs than in other ALL types (Figure S4). Comparison of the levels of *RUNX1* and the *RAG* genes within the dataset (*n* = 750) identified significant, negative correlations between *RUNX1*/*RAG1* and *RUNX1*/*RAG2* (Figure 6). This pattern was evident in the total ALL dataset with or without the t(12;21) subset, where interpretation is more complex due to the detection of *TEL-RUNX1* as well as *RUNX1* mRNAs by the *RUNX1* probe sets. While the lack of significant correlation between *RUNX1* and *RAG1* and the weaker correlation between *RUNX1* and *RAG2* in t(12;21) cells is therefore difficult to interpret, the strongly negative correlation in other ALL sets is a robust observation.

### Eμ-Myc lymphoma cells lacking *Runx1* display growth impairment and increased sensitivity to genotoxic stress and dexamethasone-induced apoptosis

**Figure 7:**
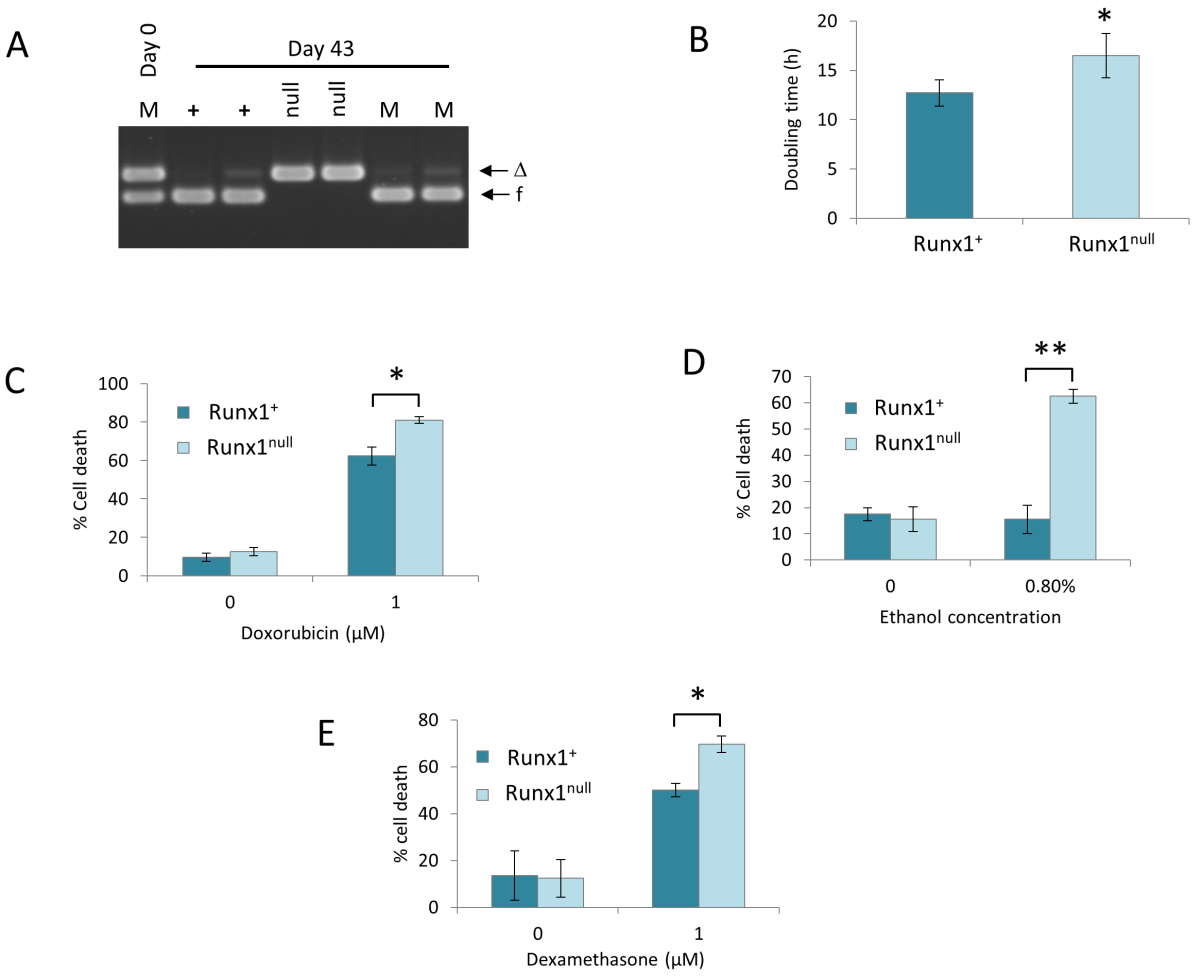
Excision of *Runx1* impairs proliferation and survival of E**μ**-Myc lymphoma cells *in vitro* **A.** *Runx1* excision PCR analysis of genomic DNA from duplicate samples of *Runx1^+^* (+) and *Runx1^null^* (null) cells or of a 1:1 mixture of these cells (M) on day 0 and after culture for 43 days. Arrows indicate *Runx1* floxed (f) and *Runx1*-deleted (Δ) bands. Results shown are from a single experiment representative of 3 independent experiments. **B.** Increased doubling time of *Runx1^null^* cells. *Runx1^+^* and *Runx1^null^* cells were plated at a density of 2×10^5^ cells/ml and cultured for 24h before counting. Doubling time was calculated as described in Materials and Methods. **C**.-**E**.Increased sensitivity of *Runx1^null^* cells to chemotherapeutic agents and oxidative stress. *Runx1^+^* and *Runx1^null^* were plated at 2×10^5^ cells/ml and treated with 1.0μM doxorubicin (C), 0.8% ethanol (D) or 1.0μM dexamethasone (E) for 24-30 hours before viability counting. Plots show mean ± SD for a single experiment carried out in triplicate and are representative of at least 3 independent experiments; * = *p* < 0.05, ** = *p* < 0.01.

Careful observation suggested that the *Runx1*^null^ cell lines grew more slowly, and this suspicion was confirmed by serial passage of clonal Eμ-Myc/*Runx1^fl/fl^* cell lines in which partial excision had been induced. Cells retaining *Runx1* consistently outgrew their null clonal siblings (Figure 7A). This disadvantage could be accounted for by the observed lengthening of doubling time in *Runx1*-deleted cells (Figure 7B) operating over the prolonged culture period. Marked differences were also noted when cells were exposed to genotoxic stresses, where *Runx1^null^* cells displayed more rapid death in the presence of doxorubicin or ethanol (Figure 7C, 7D). The effects of ethanol were particularly potent on *Runx1^null^* cells (Figure 7D), possibility reflecting the fact that ethanol elicits wider oxidative stress-induced effects in addition to DNA damage [34, 35]. In light of our previous findings that ectopic Runx1 expression suppresses glucocorticoid growth inhibition in murine fibroblasts [36] we also tested the effects of dexamethasone. Again, *Runx1*-deficient Eμ-Myc lymphoma cells displayed significantly greater induction of cell death (Figure 7E)

### Eμ-Myc lymphoma cells are tumorigenic but circulating tumor cells display increased sensitivity to dexamethasone

**Figure 8:**
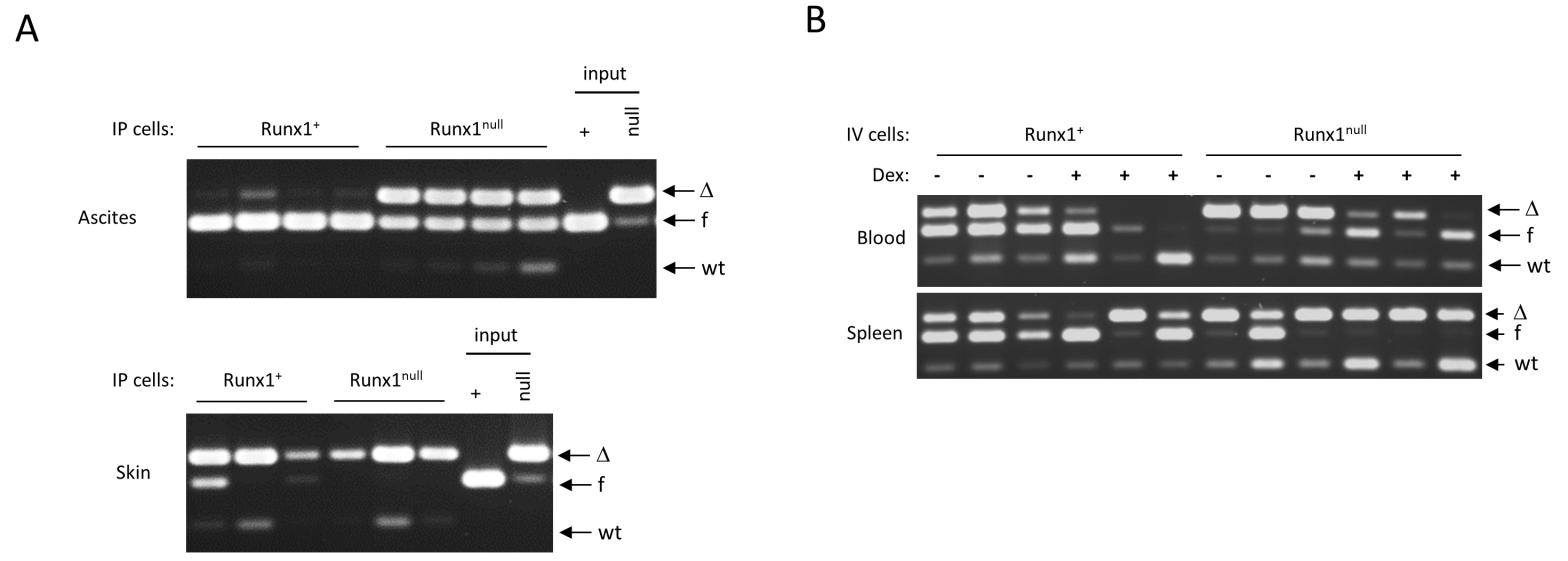
*Runx1^null^* lymphoma cells are tumorigenic but are out-competed by *Runx1^fl/fl^* cells in ascitic fluid and in the blood of in dexamethasone-treated mice **A.** *Runx1* excision PCR on genomic DNA from vehicle control (Runx1^+^) or IFNβ-treated (Runx1^null^) 3s cell line cells (“input”), and from cells from ascites fluid (upper panel) and tumors in skin (lower panel) derived from 4 mice transplanted IP with each of the input cell types. **B.** *Runx1* excision PCR on genomic DNA of cells derived from blood and spleen of 12 mice comprising 6 mice transplanted intravenously with *Runx1^+^*(3s+) and 6 with *Runx1^null^* (3s-) cells, and 3 mice from each of these groups treated with dexamethasone or vehicle control as detailed in Materials and Methods.

The unexpected survival of Eμ-Myc lymphoma cell lines after *Runx1* excision *in vitro* led us to consider whether Runx1 is required for lymphoma re-establishment *in vivo*. This was tested by inoculation of NOD-SCID/γC^null^ (NSG) mice with cell lines with and without excision of *Runx1* (Figure 8). Retrospective analysis showed that the *Runx1*-excised input cells had a small residual fraction of non-excised cells, while the non-excised input cells were virtually pure (Figure 8A). Notably, the non-excised cells appeared to have a significant advantage in cells obtained from ascites but not in tumors that arose within the skin at the injection site, where mainly excised cells were observed. As these mice were not treated with pIpC, the non-excised cells had clearly undergone spontaneously induced deletion in the skin tumor deposits. While this phenomenon may also reflect local production of endogenous IFNβ in skin e.g. by plasmacytoid dendritic cells [37] the key observation for this study is that tumors can develop *in vivo* with no detectable Runx1. This analysis also shows that there is a strong selective advantage for retention of Runx1 in cells growing in suspension in the peritoneal cavity.

We also tested the effects of administration of dexamethasone on tumor formation. The most striking observation here was in circulating cells in blood, where dexamethasone treatment had a much greater effect in eliminating excised cells compared to their non-excised counterparts (Figure 8B). This observation illustrates the fact that the growth requirements of cells *in vivo* cannot be fully elucidated *in vitro* and suggests that free cells in circulation are most dependent on Runx1 for their survival. Notably, some splenic lymphomas appeared to consist of exclusively *Runx1^null^* cells (Figure 8B, lower panel), again indicating attenuation of Runx1-dependency compared to primary lymphomas.

## DISCUSSION

Previous studies have shown that ectopic expression of the *Runx* genes as a result of retroviral insertional mutagenesis or transgenic over-expression is potently synergistic with over-expressed Myc or loss of p53 in lymphomagenesis [9–11, 38, 39]. The present study shows that basal expression of the normal *Runx1* gene is vital for maintenance of primary Myc-driven lymphoma *in vivo* and that this dependence is stronger than in normal lymphoid cells, providing evidence of oncogene addiction *in vivo*. Surprisingly, lymphoma-derived cell lines were able to proliferate indefinitely and remained tumorigenic after complete excision of *Runx1*. This is a telling observation, as it demonstrates that Runx1 is not merely a structural component of the transcriptional apparatus that confers B-cell identity and sustains viability. However, *Runx1^null^* lymphoma cells displayed growth impairment compared to *Runx1* non-excised controls and were hypersensitive to DNA damaging agents and glucocorticoids. On transplantation, cells retaining Runx1 also had a selective advantage when growing as non-adherent cells in the peritoneal cavity and as circulating cells in the blood of mice treated with glucocorticoid. These observations suggest that *Runx1^null^* cells may be partially protected by supportive sites of high cell density *in vivo*.

The ability of p53^null^ Eμ-Myc lymphoma cell lines to survive *Runx1* deletion suggested a possible functional link whereby Runx1 protects against Myc-driven induction of p53 and/or downstream responses, and becomes superfluous after loss of the intact *Tp53* allele during *in vitro* establishment. However, reintroduction of a temperature-sensitive p53 expression construct (Val135) into cell lines did not discriminate between *Runx1* positive and negative cells, which succumbed to apoptosis with similar kinetics after temperature shift with or without irradiation. It is conceivable that another change, secondary to *Trp53* allele loss, allows the established cells to survive without *Runx1*. This hypothesis is consistent with the observation that the *Trp53^null^* blast cell fraction *in vivo* showed substantial retention of the intact *Runx1* allele. However, we cannot exclude the possibility that ectopic p53 *in vitro* fails to recapitulate fully the behaviour of endogenous p53 *in vivo*. For example, we were surprised to observe a lack of induction of detectable CDKN1A/p21^Waf1^ protein expression in response to irradiation and p53 activation. This observation implies that established Eμ-Myc lymphoma cells may have lost the capacity to undergo cell cycle arrest in response to p53 induction and choose cell death as the default pathway. This phenomenon could account for the increased selection against wild-type p53 in cultured lymphoma cells compared to their *in vivo* counterparts [14].

The cell-type specific regulatory processes controlled by the Runx transcription factors are underlined by transcriptome analysis of Eμ-Myc lymphoma cells before and after *Runx1* excision, which revealed many changes that were not evident in control *Runx1^wt/wt^* lymphoma cells with inducible Cre expression. The Runx1 gene expression signature we observed in Eμ-Myc lymphoma cells was significantly enriched for regulators of lymphocyte proliferation, survival and differentiation, with changes consistent with the observed growth advantage and chemo-resistance associated with intact Runx1 expression.

The marked up-regulation of *Rag1* and *Rag2* after *Runx1* deletion is of particular interest. These genes are regulated in a complex, lineage-specific manner in T- and B-cells and undergo waves of expression during B-lymphocyte development [40]. The promoters of both genes harbour multiple Runx binding sites, while further Runx motifs are essential for the function of an intergenic Rag silencer in T-cells which is over-ridden in double positive thymocytes by an anti-silencer upstream of *Rag2* [41]. We also noted strong inverse correlations between *RUNX1* and *RAG1/RAG2* mRNA expression in a panel of human leukemias and lymphomas. This is interesting in light of evidence that driver mutations are frequently induced by aberrant RAG activity in t(12;21) TEL-RUNX1 leukemias [42] which express unusually high levels of *RAG1*, but also in other genotypes that have no known lesion in *RUNX1* [43, 44]. The weaker correlation between *RUNX1* and *RAG* expression in t(12;21) leukemias may be an artefact of the detection of mRNA for both *TEL-RUNX1* and the untranslocated *RUNX1* allele but may also be a function of direct interference with *RAG* repression by the fusion oncoprotein. Moreover, the increased incidence of T-cell lymphomas in ENU-treated chimeric mice [17] and the occurrence of apparent loss-of-function *RUNX1* mutations in a subset of ALLs [20] might be explained at least in part by dysregulation of recombinase gene expression.

We noted very little overlap with changes associated with *Runx1* deficiency in murine haematopoietic precursor cells where ribosomal biogenesis was implicated recently as a Runx1-directed function [45]. Apart from their separation in the differentiation hierarchy, Eμ-Myc cells differ from HPSCs in over-expression of Myc, a known driver of ribosome biogenesis [46]. It is therefore conceivable that the effect of *Runx1* deletion on ribosomal gene expression in HSPCs is mediated indirectly through loss of signalling to Myc. We also noted diametrically opposite effects of *Runx1* deficiency on responses to DNA damage, where HSPCs were reported to display increased resistance [47], while we noted increased sensitivity of *Runx1*^null^ Eμ-Myc cells. While this difference might be related to cell transformation, the relative permissiveness to *Runx1* deletion of the myeloid compartment in normal spleen suggests that lineage-specific factors are likely to be involved. Moreover, the increased fragility of Myc-driven lymphoma cells lacking *Runx1* indicates that targeting of Runx pathways is likely to be of therapeutic benefit in the context of Myc-driven lymphoma. We also noted little overlap with the mitotic checkpoint signature observed after knockdown of RUNX1 in Kasumi t(8;21) AML cells [31]. While lineage-specific differences may again be invoked to explain this discrepancy, the AML cells also express the RUNX1-ETO fusion protein which is likely to modulate at least some of the key promoters and enhancers vacated by RUNX1 knockdown.

The finding that basal *Runx1* activity is critical for Myc-driven lymphoma maintenance *in vivo* and that dependence is only partially attenuated in established cell lines lacking p53 is encouraging for ongoing efforts to target the *Runx* genes and their downstream effectors in cancer therapy [48, 49]. Moreover, the increased sensitivity of *Runx1*-deleted cells to components of standard chemotherapeutic regimens in current use for lymphoma therapy suggests that these may be combined with Runx inhibition for greater efficacy.

## MATERIALS AND METHODS

### Generation of transgenic crosses and animal experiments

Mx1Cre^+^/*Runx1^fl/fl^* mice [26] were crossed with Eμ-Myc mice and *p53^−/−^* mice to produce highly tumour-prone Mx1Cre^+^/*Runx1^fl/fl^*/Eμ-Myc^+^/*p53^+/−^* mice and littermate controls lacking Mx1Cre and/or Eμ-Myc. Litters were treated at two weeks of age with 8.5mg/kg pIpC injected intraperitoneally (IP; two injections two days apart) or left untreated; animals were humanely culled when they showed signs of tumour development. *Runx1^wt^* controls (Mx1Cre^+^/*Runx1^wt/wt^*/Eμ-Myc^+^/*p53^+/−^*) were treated in the same way. Adult Eμ-Myc^−^/*Runx1^fl/fl^* mice were treated with 6 injections of 600μg pIpC 2-3 days apart at 5 weeks of age. Transplantation assays were performed in NOD/SCID/γCn*^null^* (NSG) mice, which were transplanted IP or intravenously (IV) with 5 × 10^6^ *Runx1^+^* or *Runx1^null^* cells; IV-transplanted mice were treated with 20mg/kg Dexadresone^®^ IP or vehicle control daily, Monday-Friday for 3 weeks and 5-10μl blood for flow cytometric analysis of B220^+^ cell counts were removed weekly by tail-tipping. Animal protocols used in this work were evaluated and approved by the University of Glasgow Ethics and Welfare Committee and were carried out under Home Office License (approval granted September 2012, license number PPL 60/4408) as governed by the Animal Scientific Procedures Act, 1986.

### Statistical analysis

Survival curves were compared using the Wilcoxon rank sum test. All other statistical comparisons were performed using the Student's *t*-test.

### Flow cytometry and cell sorting

Phenotyping of spleen cells was performed using the following antibodies: anti-B220-phycoerythrin, anti-CD4-phycoerythrin, anti-Mac1-PerCPCy5.5 (all BD Biosciences) and anti-CD8-FITC (Serotec). Spleen cell suspensions in PBS + 0.1% BSA (wash buffer) were stained with combinations of antibodies or isotype controls for 30 minutes at 4°C, red cells were lysed using Pharmlyse (BD Biosciences) and cells were analysed using an Accuri C6 flow cytometer and Cflow Sampler software (BD Biosciences). Analysis of activated caspase 3 expression was performed by flow cytometry using the PE Active Caspase 3 Apoptosis Kit (BD Biosciences) according to manufacturer's instructions. Sorting of spleen lymphoid and myeloid cells was performed using a combination of anti-B220-phycoerythrin and anti-CD3-phycoerythrin to identify lymphoid cells and anti-Mac1-PerCPCy5.5 to identify myeloid cells. Sorting of blast cells from primary lymphomas was performed by staining cells with anti-B220-phycoerythin and anti-CD45-FITC; in the B220^+^ population, blast cells were identified using CD45 and SSC as reported in [27]. All sorting was performed using a FACSAria (BD Biosciences).

### Genomic DNA extraction and PCR

Genomic DNA was extracted from cell lines using the DNeasy kit (Qiagen) and from tissues using the illustra Nucleon BACC2 DNA extraction kit (GE Life Sciences). Determination of DNA concentration was carried out using a Nanodrop 2000 (Thermo Scientific, Walatham, MA. USA). Analysis of *Runx1* excision in primary cells/tissues and cell lines was carried out using previously described primers and cycling conditions [50]; master mix was prepared with 2x ReddyMix, 1.6μM each primer and 10ng template DNA. Validation of this assay for quantitative determination of *Runx1* excision was performed by analysing excision in standards containing mixtures of 0-100% excised cells. PCR for *p53* wild-type and null alleles was performed using 20ng template DNA, 2x ReddyMix (ThermoFisher Scientific) and primers directed against p53 intron 4 (WP53: GTGTTTCATTAGTTCCCCAC), exon 5 (UP3: ATGGTGGGGGCAGCGTCTCA), and the null allele (NP5: CGGTCTTGTCGATCAGGATG); cycling conditions were 5 minutes at 94°C, 35 cycles of 1 minute at 94°C, 1 minute at 55°C and 1 minute at 72°C, then 7 minutes at 72°C, generating PCR products of 242bp (p53 wt allele) or 470bp (p53 null allele). All PCR products were separated on a 1.5 or 2% agarose gel and visualised with ethidium bromide and UV transillumination. Densitometry was carried out using ImageJ software (http://imagej.nih.gov/ij/); images shown were adusted only for contrast.

### Cell culture, constructs and retroviral transductions

The 3s, 6s and 44s cell lines were established from spleen tissue from primary lymphomas in Mx1Cre^+^/*Runx1^fl/fl^*/Eμ-Myc/*p53^+/−^* transgenic mice and grown in RPMI 1640 supplemented with 10% FCS, 100U/ml penicillin and streptomycin, 2mM L-glutamine and 50μM 2-mercaptoethanol (complete RPMI; all reagents from Life Technologies). Paired *Runx1^null^* and *Runx1^+^* cells were created from *Runx1^fl/fl^* parental cell lines by treatment with 5-50U/ml IFNβ (R&D Systems) or vehicle control (PBS+0.1% BSA); *Runx1* excision in IFNβ-treated lines was confirmed by *Runx1* excision PCR. The p53^V135^-GFP was constructed by ligating the 1.4kb EcoR1 fragment containing murine p53^V135^ [51] into the Mig-R1 vector [52]. Viral supernatants were prepared following transient transfection of GP86+E or 293T cells respectively and used to infect 3s cells as described previously [53]. Infected cells were sorted for GFP^+^ cells. Doubling time assays were performed in 12-well cell culture plates with 2×10^5^cells/ml and counted 24 hours after initiation of culture; doubling times were calculated using the formula T_d_ = t_i_ × (log(2)/log(q_2_-q_1_)) where T_d_ = doubling time, t_i_ = incubation time, q_1_ = number of cells at start of assay and q_2_ = number of cells at end of assay. Competition assays were performed by mixing equal numbers of excised and non-excised cells; cells were cultured in duplicate in complete RPMI, passaging three times per week, returning 10^5^ cells to 10ml culture at each passage; *Runx1* excision in the culture was monitored weekly by *Runx1* excision PCR. Ethanol, doxorubicin (Stratech Scientific #S1208 10mM in DMSO) or dexamethasone (Sigma D2915) treatment of pairs of excised and non-excised cell lines was carried out in 12-well plates using 2×10^5^ cells/ml and with the addition of ethanol (0.8%), doxorubicin (1.0μM) or dexamethasone (1.0μM). Single cell cloning of cell lines was performed by partially excising *Runx1* by sub-optimal IFNβ treatment and sorting single cells into 96-well plates in complete RPMI; growing clones were transferred to larger culture dishes until sufficient cells to analyse were obtained.

### RNA extraction and microarray analysis

Total RNA was isolated by RNeasy kit (Qiagen, Manchester, UK) from three cultures each of established lymphoma lines 3s (Mx1Cre^+^/*Runx1^fl/fl^*/Eμ-Myc^+^/*p53^+/−^*) and 30s (Mx1Cre^+^/*Runx1^wt/wt^*/Eμ-Myc^+^/*p53^+/−^*), treated with and without IFNβ to excise endogenous *Runx1* (Figure 4A). RNA was tested for quality on the Agilent 2100 Bioanalyser (Agilent Technologies, Stockport, UK) and NanoDrop 2000 (Thermo Scientific, Walatham, MA. USA) before screening against Affymetrix GeneChip Mouse Transcriptome Array 1.0 (High Wycombe, UK, 2014) by ATLAS Biolabs (Berlin, Germany) according to standard protocols. RMA normalisation followed by probe annotation and statistical analysis to generate *p*-values and fold changes was performed using Partek Genomics Suite 6.6 (Partek Inc., St.Louis, MO, USA). Microarray data are available at the Gene Expression Omnibus (GEO) repository, accession number GSE78001.

### Quantitative real-time PCR

cDNA was prepared from 1μg aliquots of RNA using a Quantitect Reverse Transcription kit (Qiagen) and diluted 1 in 20 in DEPC-treated water to give a working stock. For quantitative real-time PCR, 12.5ng aliquots of cDNA were amplified in triplicate on an ABI 7500 real-time PCR system using Power SYBR Green PCR master Mix (Thermo Fisher Scientific, UK), and primers for murine *Bcl11a, Cd55, Daf2, Il7r, Nckap1, Ptpn22, Prkcb, Rag1, Rag2* or endogenous control *18S rRNA* (Qiagen QuantiTect Primer Assays). Relative quantification was carried out and calibrated to vector control samples where appropriate. Data were analysed using the standard software for the ABI 7500 real-time PCR system.

### Western blotting and antibodies

Preparation of whole cell protein extracts was performed as described previously [54]. Samples equivalent to 50μg of protein (Bio-Rad protein assay) were resolved on 8-12% SDS polyacrylamide gels and transferred to enhanced chemiluminescence (ECL; Fischer Scientific), nitrocellulose membranes. The antibodies used were α Runx1 (#8229 New England Biolabs), α p53(FL393), α p21^WAF1^, α Actin (sc-6243, sc-471 and sc-1616, Santa Cruz Biotechnology) and α CDKN2A/p19ARF (ab80, Abcam). Positive controls were as follows: Runx1 (NIH3T3 transduced with pBabeRunx1 P1 [15], p53 and p21^WAF1^ (UVC-treated wild type MEF extract), p19^ARF^ (SV3T3 cell extract).

## CONCLUSIONS

Primary lymphoma cells from Eμ-Myc mice show evidence of addiction to *Runx1 in vivo*, but become permissive for deletion *in vitro*. Loss of p53 function appears to be necessary but not sufficient for this process. In this context Runx1 controls a network of genes involved in lymphocyte proliferation, survival and differentiation, shedding light on its dualistic behaviour in lymphomagenesis. While the ability of Myc-driven lymphoma cells to grow in the absence of Runx1 is surprising, their impaired proliferation and increased chemo-sensitivity validates Runx1 function as a candidate target in future combination therapies.

## SUPPLEMENTARY MATERIALS FIGURES


