# Supplementary material for: Addiction to Runx1 is partially attenuated by loss of p53 in the Eμ-Myc lymphoma model
Source: Oncotarget. 2016 Apr 2;7(17):22973–87. doi: 10.18632/oncotarget.8554 (PMC5029604; doi:10.18632/oncotarget.8554)
Supplement: Supplementary file 1 [file oncotarget-07-22973-s001.pdf]

Addiction to *Runx1* is partially attenuated by loss of p53 in the Eμ-Myc lymphoma model

Supplementary Material

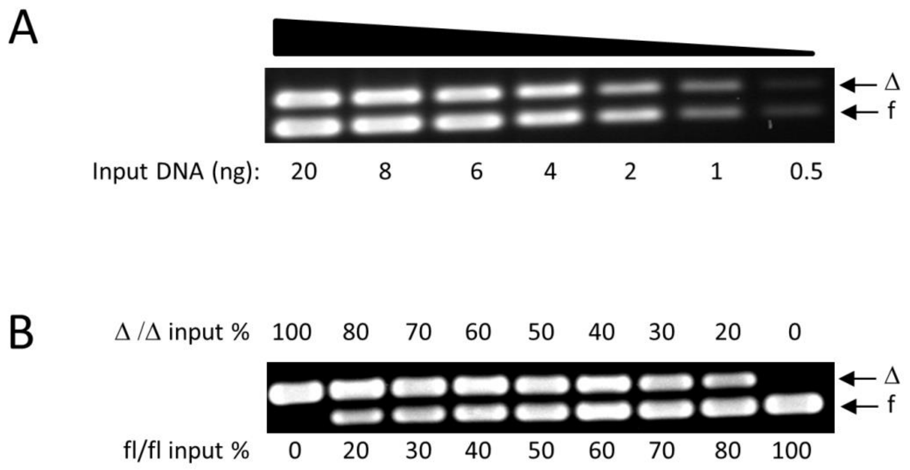

Figure S1

Validation of PCR assay to estimate relative quantities of excised and non-excised *Runx1*$^{fl/fl}$ alleles in tissues and cell lines. (A) *Runx1* excision PCR was performed on a mixture of equal amounts of DNA from cell lines with completely *Runx1*-excised and non-excised genomic DNAs (Figure 1D). The total quantity of template DNA is shown above the lanes. (B) *Runx1* excision PCR was performed on 20ng template DNA composed of mixtures of varying proportions of *Runx1*-excised and non-excised genomic DNA.

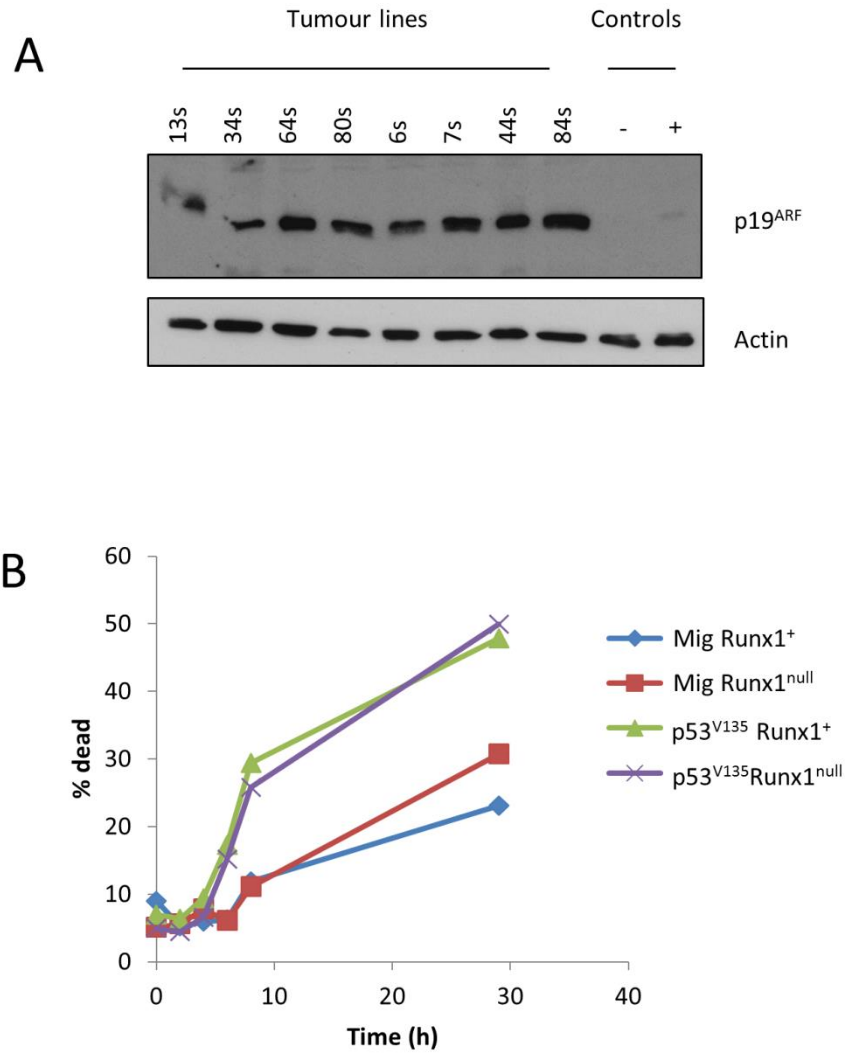

Figure S2

Additional analyses of lymphoma cell line p19 expression and effect of Runx1 on apoptosis of p53 addback cells. (A) Western blot analysis of total protein extracted from a panel of independent lymphoma cell lines derived from lymphomas in Mx1Cre^{+}/*Runx1*$^{fl/fl}$/Eμ-Myc^{+}/*p53*$^{+/-}$ mice. Extracts were probed with antibodies to p19^{ARF}. Actin was used as a loading control. Positive controls are listed in Materials & Methods and used in all subsequent analyses. (B) Paired Runx1^{+} and Runx1^{null} p53 addback cell lines were grown at 32°C to activate temperature sensitive p53 and stained for intracellular activated caspase 3 after 2, 4, 6, 8 and 30h incubation. The percentage of cells expressing activated caspase 3 was determined by flow cytometry.

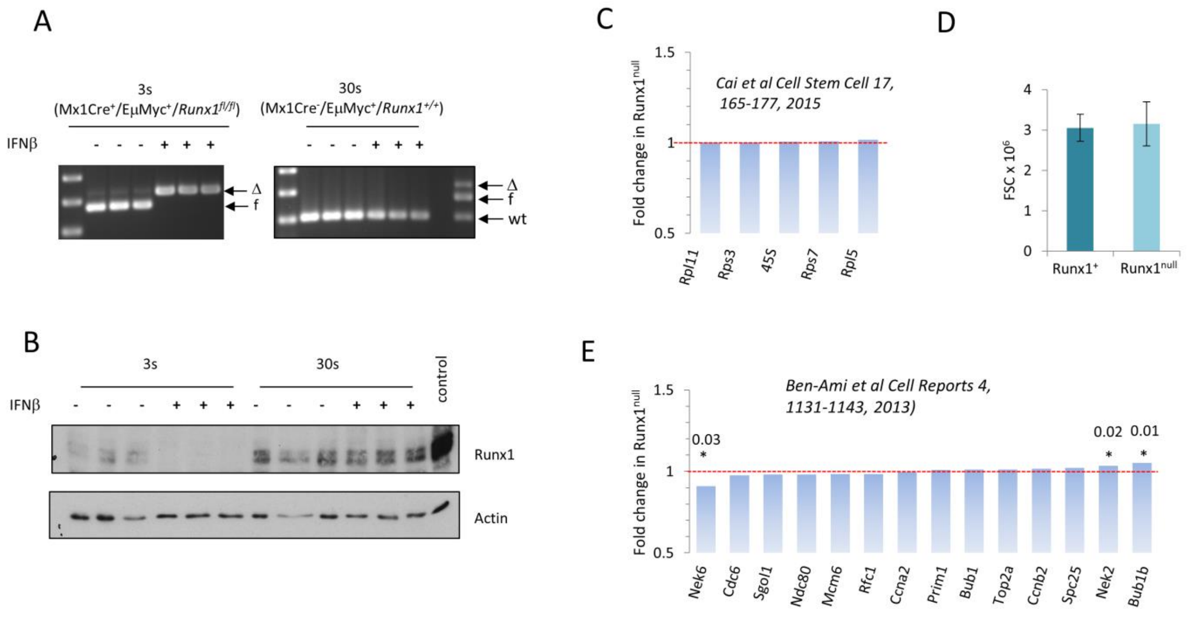

Figure S3

Supplementary analyses of samples and data analysed by gene expression microarray (Figures 4, 5). The biological replicates used for RNA preparations were shown to have the expected genotype by PCR analysis (A), where only *Runx1*$^{fl/fl}$ allele was affected by IFNβ treatment and by western blot analysis for Runx1 expression (B) which confirmed the loss of Runx1 protein from treated *Runx1*$^{fl/fl}$ cells and the lack of change on *Runx1*$^{wt/wt}$ 30s cells. Direct analysis of Runx1 gene signatures reported in other studies were analysed directly by comparing the expression of key target genes in excised and non-excised 3s cells. (C) No significant changes were observed in genes that showed major changes in the ribosomal biogenesis signature observed by Cai and co-workers. Also, unlike this study we noted no difference in cell size after excision of Runx1. (D) Size of Runx1^{+} (3s+ cells) and Runx1^{null} (3s-) cells was compared by analysis of flow cytometric forward scatter (FSC). Plot shows mean FSC ± SD from 16 individual measurements for each of Runx1^{+} and Runx1^{null} from 5 separate experiments. (E) Key genes representing a mitotic checkpoint signature observed in Kasumi AML cells after knockdown of RUNX1 did not show similar changes in *Runx1* excision Eμ -Myc lymphoma cells. While some significant changes were noted, these were not fully concordant with the published study, while most genes in the signature showed no significant change in our study.

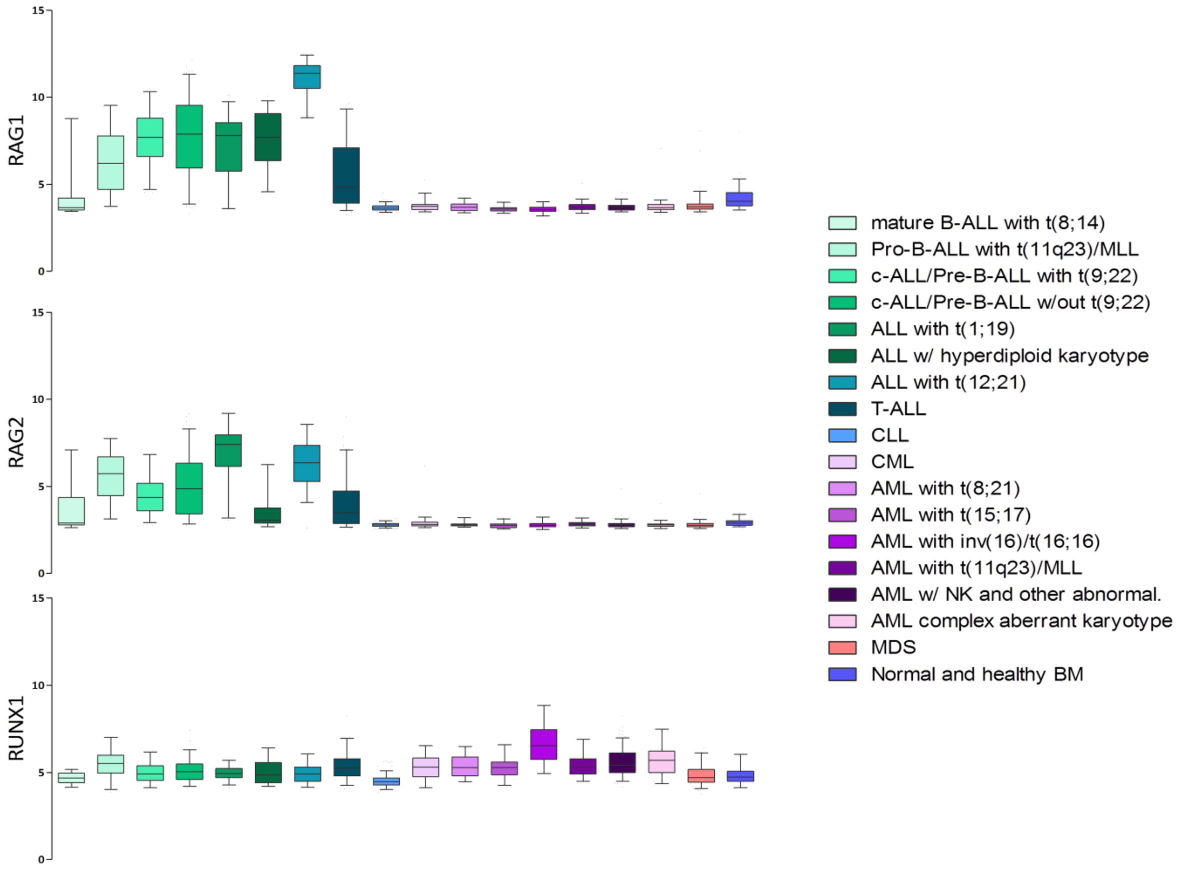

Figure S4

Gene expression plots across a panel of hematopoietic cancers and normal tissues, from the MILE dataset (ref [33]), for (A) *RAG1*, (B) *RAG2* and (C) *RUNX1*. Data are presented as box and whisker plots with the box representing the upper to lower quartiles, the median shown by the line and the whiskers representing 5-95% percentile. The legend shows the disease subsets analysed.
